# Global research trends and prospects on the first-generation college students from 2002 to 2022: a bibliometric analysis via CiteSpace

**DOI:** 10.3389/fpsyg.2023.1214216

**Published:** 2023-07-27

**Authors:** Li Fei, Xizhen Kang, Weiming Sun, Boxiang Hu

**Affiliations:** ^1^School of Marxism, Nanchang University, Nanchang, Jiangxi, China; ^2^The First Clinical Medical College, Nanchang University, Nanchang, Jiangxi, China; ^3^Department of Rehabilitation Medicine, The First Affiliated Hospital of Nanchang University, Nanchang, Jiangxi, China

**Keywords:** first-generation college students, bibliometric analysis, CiteSpace, research trends, Web of Science

## Abstract

**Objectives:**

To review literature related to first-generation college students, the paper aims to outline the research direction, identify prominent research topics and frontiers, report on current research trends, and offer valuable insights and fresh perspectives for future advancements in the field, utilizing CiteSpace.

**Methods:**

CiteSpace is a citation visualization software designed to analyze the scholarly literature and uncover potential knowledge within it. This study retrieved articles related to first-generation college students from 2002 to 2022 from Web of Science Core Collection database. After collecting the data, CiteSpace V.6.1.R3 (64-bit) was used to perform analyses on various aspects, including annual publication output, top cited journals, country and institutional affiliations, prominent authors, cited references, and keywords. The data was visualized using tools such as knowledge maps, collaborative network analysis, cluster analysis, and strongest citation burst analysis.

**Results:**

We obtained a total of 471 articles on first-generation college students. The number of publications annually is increasing, and the number of publications generally shows an upward trend, especially in 2017–2021 with a sharp growth. The United States has the most articles on this topic (395 articles), and it is also the most authoritative and influential country (with a centrality of 0.93). Followed by South Africa (14 articles) and Germany (14 articles), The top 10 cited journals and institutions are predominantly from the United States. When analyzing the top cited references and authors, the research consistently highlights the academic achievement and engagement of first-generation college students.

**Conclusion:**

This study analyzed the current situation of first-generation college students field via CiteSpace, then identify the research hotspots and frontiers on first-generation college students. Current global trends in first-generation college students researches and the growing public awareness of academic performance and equality suggest that first-generation college students researches will grow in popularity with further breakthroughs.

## 1. Introduction

First-Generation College Students (FGCS) are often defined as a student whose neither parent has completed a 4-year undergraduate education anywhere in the world, or whose parents did not graduate. Or a student who is living with and being raised by only one parent, and whose only parent has not completed a bachelor’s degree (Ortega, 2018; Covarrubias et al., 2020; DiGuiseppi et al., 2020), he/she is the first generation in their family to attend college and receive higher education. If an applicant’s older sibling has graduated from a four-year college, the applicant is still a FGCS because the applicant’s siblings are of the same generation. In contrast, having at least one parent with a college degree is called continuing-generations college students (CGCS; Jones and Schreier, 2022). According to statistics approximately 24% of full-time undergraduate students at four-year institutions are first-generation students, the majority of FGCS are white (about 46%), and then Hispanic or Latino (about 25%), black or African American (about 18%; Romanelli, 2020). In addition, more first-generation students identifying as racial/ethnic minorities, coming from low-income families, and having some degree of financial barriers to higher education. FGCS include a diverse group with intersecting identities connected to gender, race, ethnicity, sexual orientation and the status of socioeconomic (Mason et al., 2022). FGCS are an increasingly visible group that has received attention from universities in recruitment and retention, as well as from researchers trying to understand their unique educational experiences (King, 2021). Discussions focused on FGCS and their difficulties and achievements have become universal on most college campuses. Increasingly academic institutions and researchers begin to pay attention to and continue their analysis of this group.

Studies (Gaudier-Diaz et al., 2019; Noel et al., 2021) show that first-generation college students had higher levels of depression and trait anxiety, worse academic performance than the continuing-generation students. Due to grow up with college-educated family members, it could increase expectations for a degree for continuing—generation students (Mcdossi et al., 2022). By contrast, first-generation students aren’t exposed to the same family dynamics and go to college more in pursuit of a higher-paying job (Ellis et al., 2019). In addition, increased overall stress, lack of knowledge about the college system, differences in college preparation, limited access to advanced courses due to attending an underfunded high school, different expectations for college based on family experiences, and personal experiences may also contribute to higher depression and trait anxiety among first-generation college students (Amirkhan et al., 2022). In addition to affecting academic performance, first-generation college students are more likely to drop out of school than their next generation peers (Hood et al., 2020). Hood et al. (2020) found a reduced sense of belonging, difficulty integrating into university life, lower academic self-efficacy, greater fear of academic failure and more uncertainty, and greater concern about specific active learning practices.

A growing number of studies have proposed solutions to the physical and psychological problems of FGCS. Interventions designed to address various aspects of motivation and anxiety may help improve the academic performance of FGCS (Gaudier-Diaz et al., 2019). Indeed, formulating intervention strategies to help this disadvantaged group of students succeed academically is critical to the diversity of the education field. Difference-education can increase the comfort level of social group differences. Townsend et al. implemented difference-education intervention at two universities and found that nearly 4 years later, first-generation students who received the difference-education intervention were more likely to have higher grades and more honors than students who did not receive difference-education (Townsend et al., 2021). Their findings provide the first evidence that teaching contextual theories of difference to first-generation students can have long-range academic benefits that can last through graduation. Furthermore, Fabiano, (2022) draws upon personal experience as a first-generation college student to offer advice to others in similar situations. This includes tips on selecting a suitable major, adjusting to college life, building a strong support system, pursuing research opportunities, participating in outreach activities, promoting inclusive experience, managing academic coursework alongside other commitments, and seeking out scholarship opportunities. Such guidance provides valuable direction and support for the first-generation college students.

Bibliometrics is a quantitative approach to analyzing scholarly publications, which has been applied in many areas of research to assess the patterns of authors, institutions, journals, countries and keywords et al. associated with particular types of publication (Liang et al., 2017). It is also a good choice for identifying research trends and knowledge gaps of a research field over time (Wu et al., 2021). CiteSpace, as one of visualization analysis software, is widely used to explore the research hotspots, research frontiers, knowledge base, main authors and institutions of a research field, as well as to help predict the future direction of a research field (Chen et al., 2022). However, there is a lack of summary and evaluation of the characteristics of the literature, research directions, depth of research, and research hotspots in FGCS research. Therefore, there is a need to determine the current state of FGCS to inform future researches.

In this study, it was aimed to analyze the global research trends and prospects on the FGCS last two decades years, CiteSpace was applied to make a bibliometric analysis of related articles collected from Web of Science Core Collections database from 2002 to 2022. Patterns of research publications in this field were mapped to author, institutions, journals, countries, keywords, references, research themes, research hotspots and emerging research areas regarding FGCS.

## 2. Methods

### 2.1. Data source

Web of Science (WoS) is the premier research platform for information in the natural sciences, social sciences, arts and humanities, and the independent global citation database for the world’s most trusted publishers (Luo et al., 2021; Zhou et al., 2021). Moreover publications included in the Web of Science Core Collection (WoSCC) are considered to be an essential component of the research process (Wu et al., 2021). For this study, we chose WoSCC database as our primary data source.

### 2.2. Search strategy

**Figure 1:**
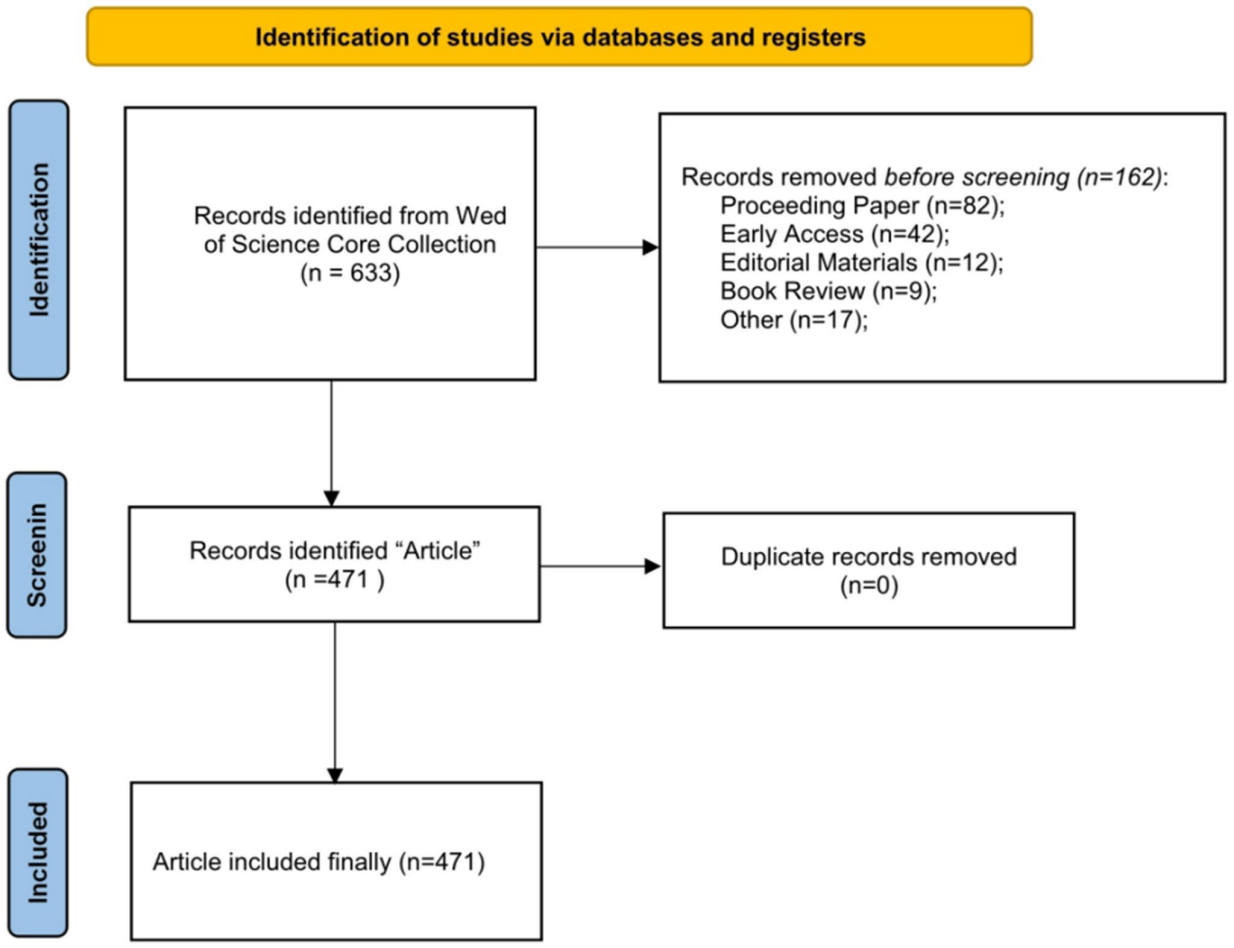
The flowchart of included publications in this study.

All the data was obtained from WoSCC on December 18, 2022. The data retrieval strategy consisted of the following four parts: (i) Topic = “first-generation college students” or “first-generation university students” or “first-generation students” or “first-generation graduates” or “first-generation undergraduates” or “First-Gen” or “first-generation to college” or “first-generation to university”; (ii) Document Type = article; (iii) Language = unlimited; (iv) Publication Year (custom year range) = 2002–2022. Full records and their corresponding cited references were downloaded in plain text format for further analysis. The flowchart is shown in Figure 1.

#### 2.2.1. Inclusion criteria

Inclusion criteria were: (i) articles on FGCS, including basic and clinical research; (ii) articles published from 2002 to 2022; and (iii) articles retrieved from the WoSCC. There were no limitations placed on the language of study reviewed.

#### 2.2.2. Exclusion criteria

Exclusion criteria were: (i) articles collected by hand and telephone or newsletters, notices, announcements, calls for papers and conference papers; (ii) articles not officially published; (iii) conference abstracts and proceedings, corrigendum documents; (iv) the same study or duplicate publications; and (v) unrelated articles.

### 2.3. Analysis tool

CiteSpace, a bibliometric analysis software developed by Chen (2004), is a citation visual analysis tool that enables the exploration of knowledge potential in scientific literature and gradually evolves under the background of scientometrics and data visualization (Gao et al., 2022). This study utilized CiteSpace V.6.1.R3 (64-bit) to analyze relevant research on FGCS, The objective was to provide evidence-based support for educators and researchers, gain insights into the current state and trends in the field, and generate new ideas for future development.

### 2.4. Data analysis

In this study, CiteSpace software was utilized to identify the citation bursts across various dimensions, including research publication year, author, research institution, journal, country, keywords, and hotspots. CiteSpace can generates a visual knowledge graphs consists of nodes and links, where nodes represent different elements such as institutions, authors, countries, and cited references, and links between nodes indicate collaborative or co-cited relationships. The size of nodes reflects their frequency or amount, while different colors represent different years, with darker colors indicating earlier years and lighter colors indicating recent years. Additionally, purple circles denote centrality. Nodes with high centrality are often regarded as turning points or pivotal points in the field (Liu et al., 2019).

## 3. Results

### 3.1. Annual publication analysis

**Figure 2:**
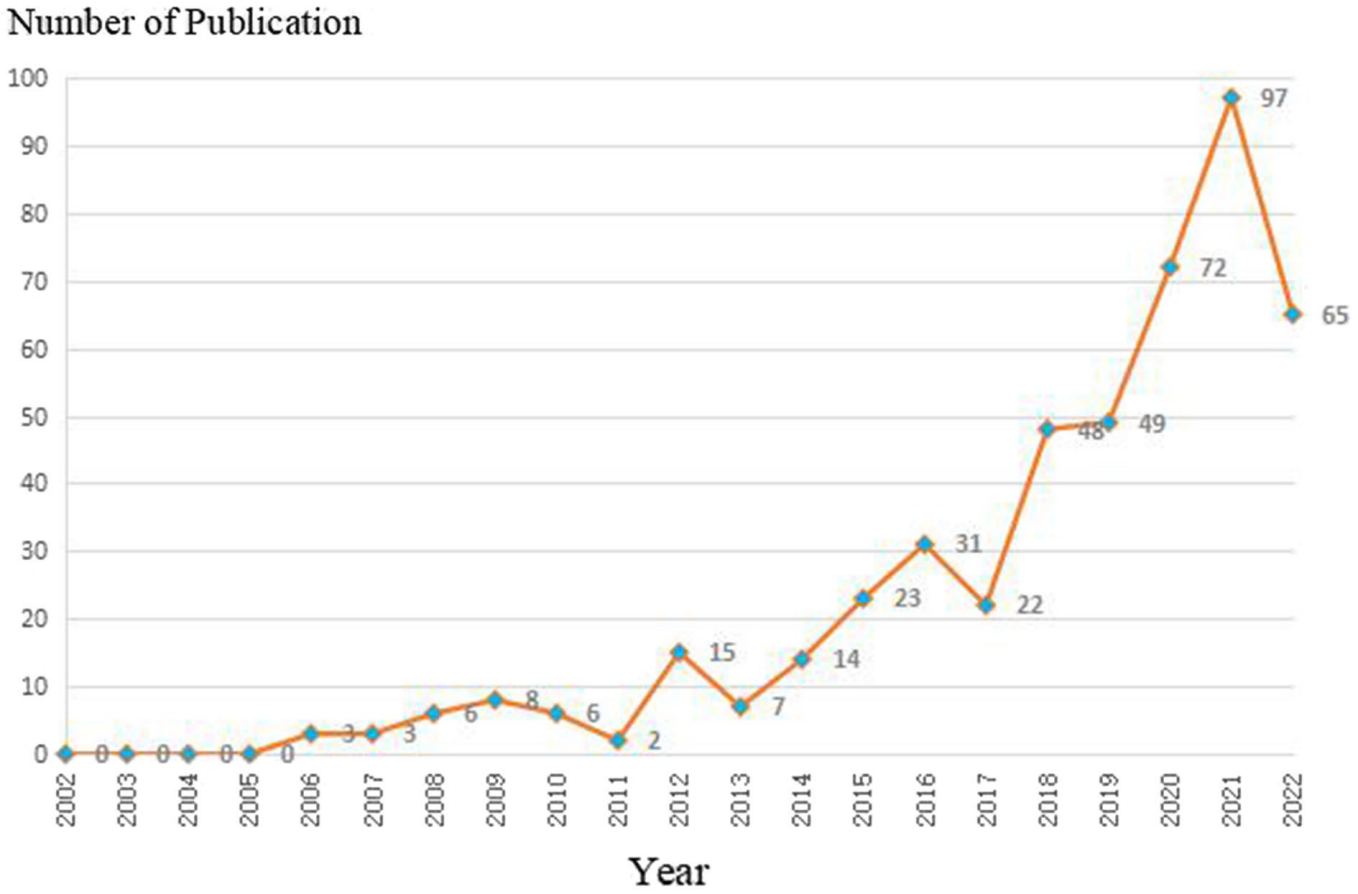
The number of annual publications and growth trends of First-Generation College Students (FGCS) from 2002 to 2022.

Utilizing the search strategy and retrieving a total of 471 inclusion criteria publications, and the number of articles published each year was shown in Figure 2. The year 2006 is the first qualified article of the first-generation college students. However, it must be admitted that there were also publications published before that, the number of which was very small and did not conform to our research inclusion criteria. As we can see, the number of articles published in FGCS can be said to be relatively small in the decade from 2002 to 2011, with no more than ten articles per year, and even no articles posting before 2006. From 2011 onwards, the number of FGCS publications started to rise. We can see two big growths, from 2013 to 2016 and 2017 to 2021, respectively. Interestingly, the number of articles published in the last 5 years is greater than the cumulative number of articles published from 2002 to 2017. Furthermore, it is obvious that this is still a very young field, because of most of the papers were published in the last decade or so.

### 3.2. Cited journals analysis

**Figure 3:**
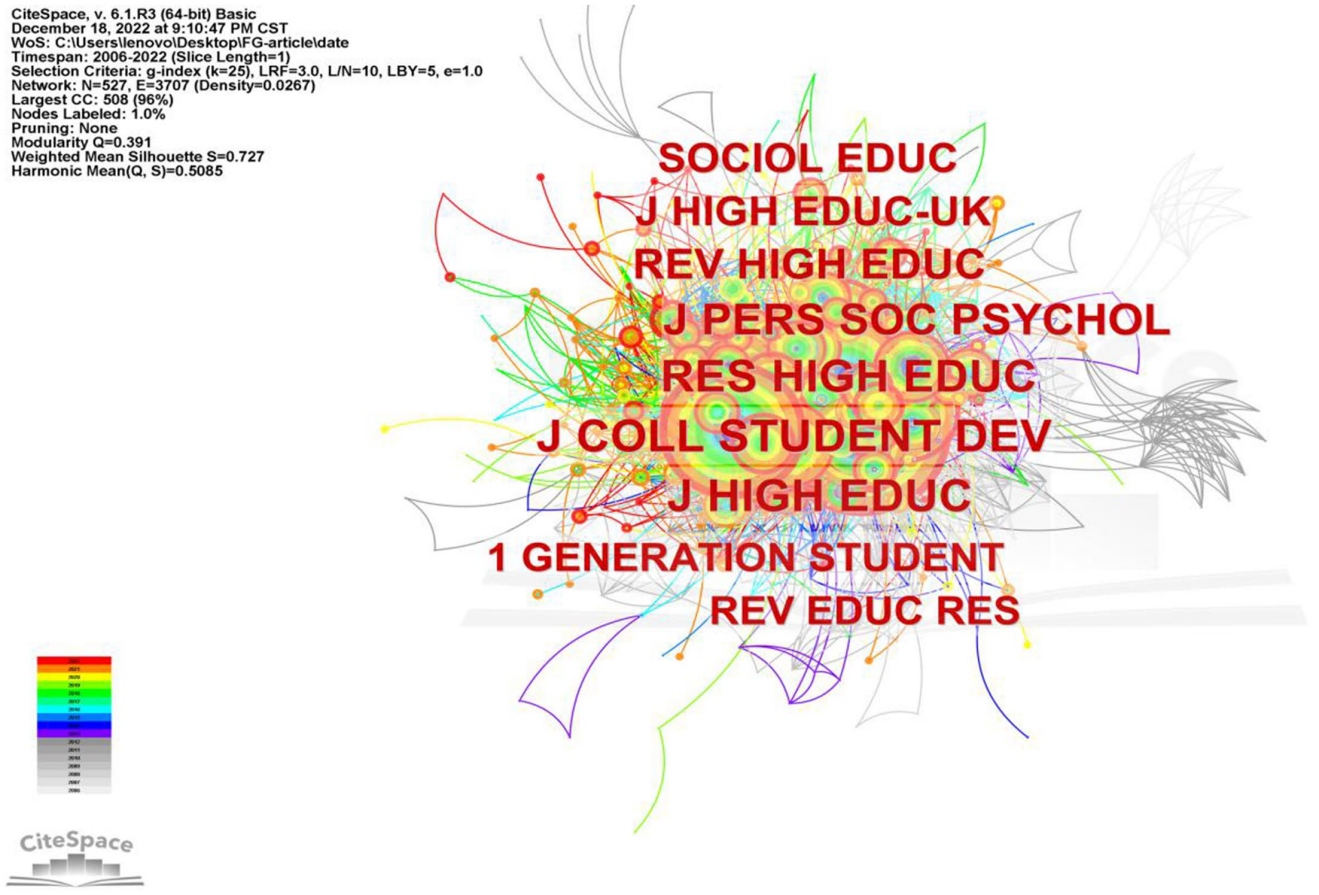
Co-occurrence map of cited journal of FGCS from 2002 to 2022.

**Figure 4:**
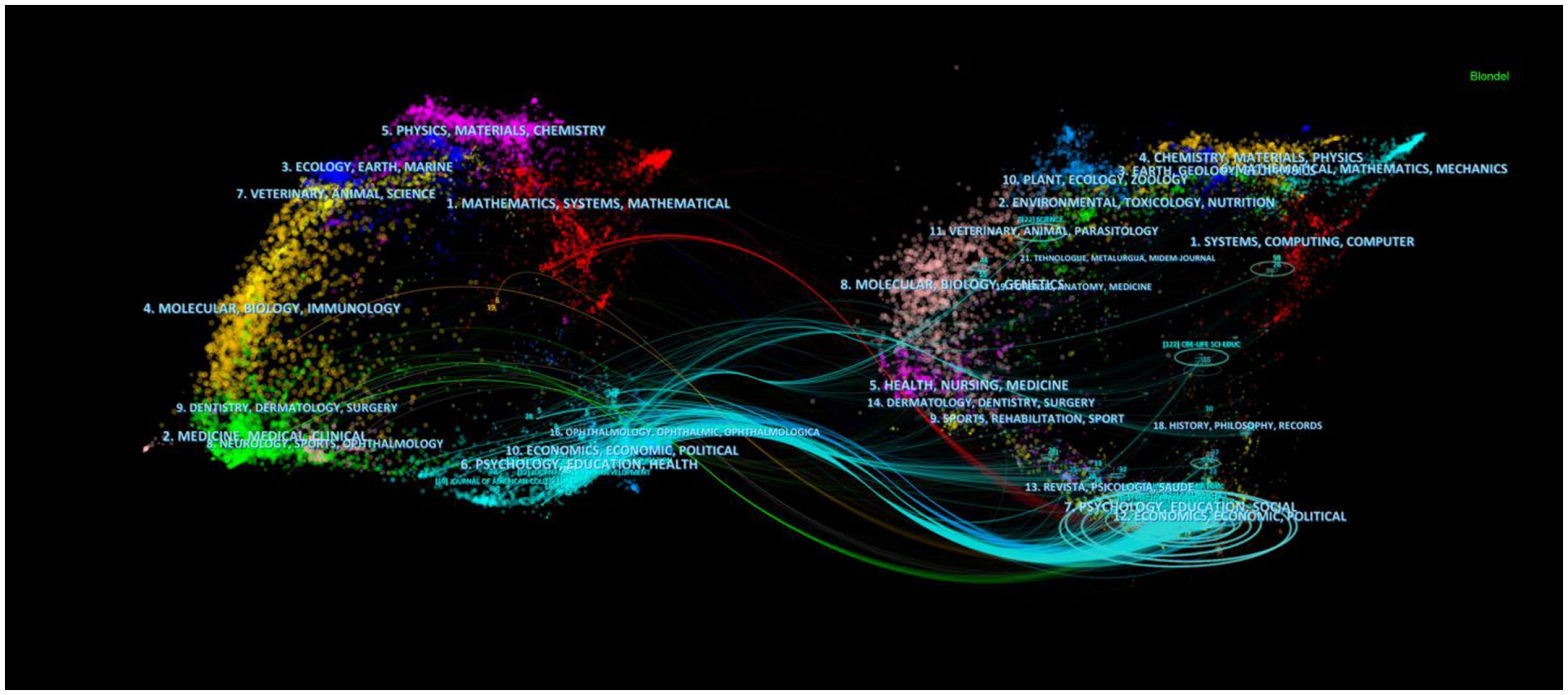
A dual-map overlay of the journals of FGCS research.

By analyzing the journals that are cited in the field of FGCS, we can gain insights into the key sources of knowledge distribution and efficiently locate relevant information. In this study, we analyzed all articles on FGCS published from 2002 to 2022 using 1-year slices and categorized the cited journals as nodes, resulting in a distribution map of cited journals with a merged network which consisted of 527 nodes and 3,703 links, as shown in Figure 3. Nodes represent journals and lines are used to describe the relationship between journals. Nodes with higher frequency are usually considered to be important nodes that have a greater impact on the development of a scientific field. The thicker the line between two nodes indicates a closer relationship. Figure 4 displays a dual-map overlay of journals, showing the distribution of relationships between cited journals on the left and citing journals on the right, and the colored paths connecting the journals represent citation relationships. Many journals are education-related specialized journals, other remaining journals are psychological and specialized sociology journals. The top ten cited journals in this area of study are shown in Table 1. Research in higher education ranking first with 207 publication, followed by Journal of college student development with 205 publications and Journal of higher education with 187 publications. The journal with the highest image factor is Journal of personality and social psychology, and it is also the fourth most published journal. Most of the top ten journals listed by the number of are located in the United States.

**Table 1:** The top ten cited journal and centrality in the research field of First-Generation College Students (FGCS) from 2002 to 2022.

Ranking	Cited journal	Frequency	Centrality	IF
1	Research in Higher Education	207	0.05	2.615
2	Journal of College Student Development	205	0.04	1.478
3	Journal of Higher Education	187	0.06	2.508
4	Journal of Personality and Social Psychology	164	0.08	5.919
5	Review of Higher Education	134	0.10	1.023
6	Journal of Higher Education (UK)	114	0.01	0.596
7	Sociology of Education	112	0.06	1.711
8	Review of Education Research	93	0.02	3.897
9	Journal of Counseling Psychology	91	0.04	2.049
10	American Education Research Journal	84	0.04	0.333

### 3.3. Countries analysis

**Figure 5:**
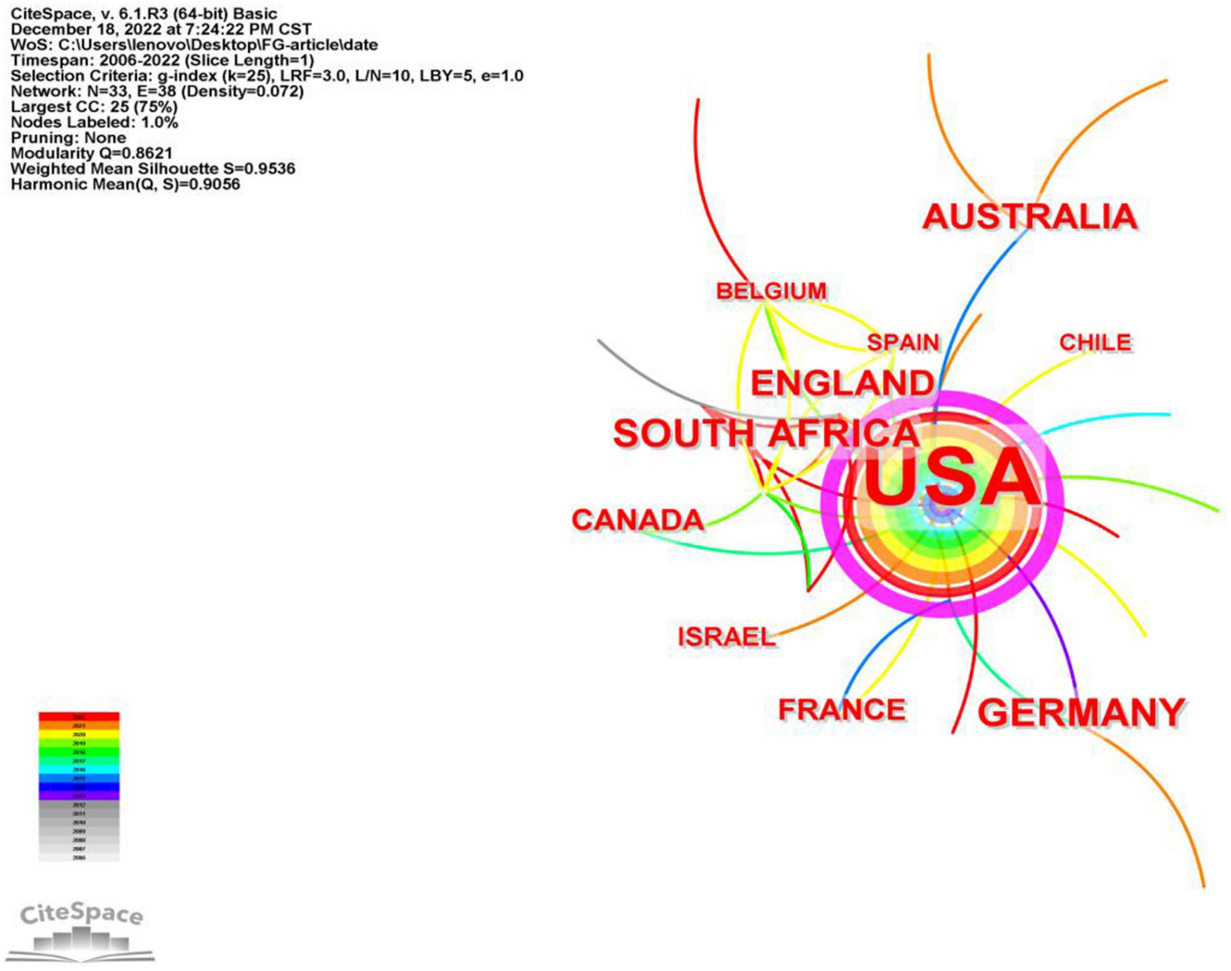
Co-occurrence map of countries of FGCS from 2002 to 2022.

In order to investigate the relationships between articles published in different countries, we conducted an analysis of all articles on FGCS published from 2012 to 2022, using 1-year slices. This analysis generated a country specific distribution map, as depicted in Figure 5, which consists of a merged network of 33 nodes and 38 links. The nodes represent countries, and the lines depict the relationships between them. The size of the nodes reflects the number of publications, with larger nodes indicating higher publication productivity. The top ten most productive countries in the field of FGCS are presented in Table 2. As we can see, the United States has the highest publications productivity (395), which is also the only country with more than 100 articles and is pulling away from other countries by a wide margin. Then followed by South Africa, Germany, England and Australia. Unfortunately, China is in 12th place with a total of 3 articles, failing to squeeze into the top ten list. There is no deny that the United States is still the bull in this field, it is difficult for other countries to catch up.

**Table 2:** The top ten countries and centrality in the research field of FGCS from 2002 to 2022.

Ranking	Countries	Frequency	Centrality
1	United States of America	395	0.93
2	South Africa	14	0.07
3	Germany	14	0.11
4	England	11	0.15
5	Australia	11	0.18
6	Canada	7	0.00
7	France	6	0.02
8	Israel	5	0.00
9	Belgium	4	0.09
10	Spain	4	0.00

### 3.4. Institutions analysis

**Figure 6:**
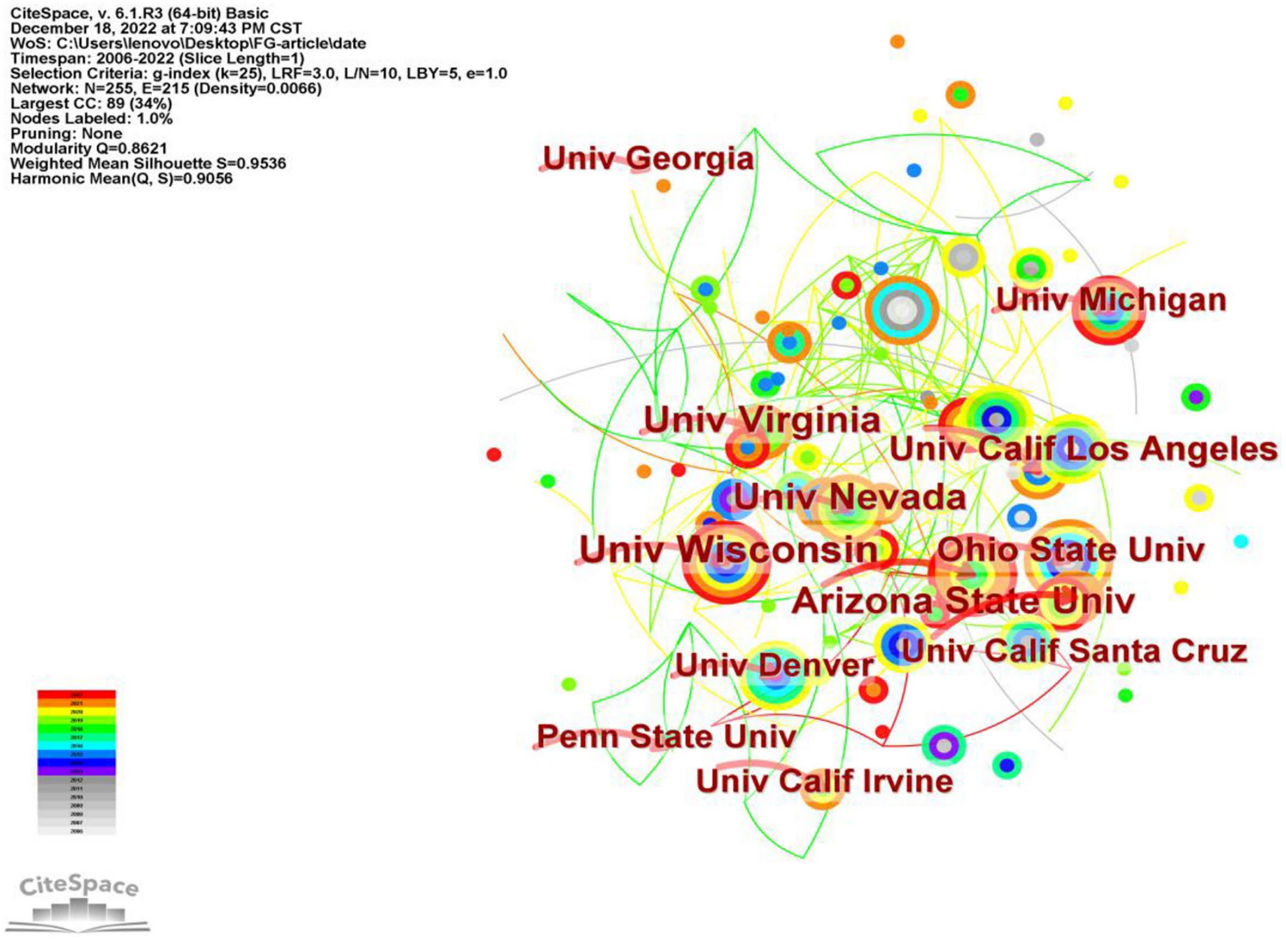
Co-occurrence map of institutions of FGCS from 2002 to 2022.

We used CiteSpace to generate a network map to explore collaborative relationships between institutions and identify influential institutions in the field of FGCS. By setting institution as the node type, we obtained a distribution map of institutions, which depicted a merged network consisting of 255 nodes and 215 links, as illustrated in Figure 6. Nodes represent institutions and lines are used to describe the relationship between institutions. Nodes with higher number of published papers are usually considered to be important institutions that have a greater impact on the development of a scientific field. The thicker the line between two nodes indicates a closer relationship. The top ten most productive institutions in this area of study are shown in Table 3. University of Wisconsin tops all institutions with 13 publication, then Arizona State University, University of Virginia and University of Nevada in the second place with 11 publications, and University of California, Los Angeles and Ohio State University hold the third place with 9 publications. Most of the top ten universities are located in the United States, which demonstrates the country’s position in the field. In addition, we can also find that the major institutions are very closely linked.

**Table 3:** The top ten institutions and centrality in the research field of FGCS from 2002 to 2022.

Ranking	Institutions	Frequency	Centrality
1	University of Wisconsin	13	0.07
2	Arizona State University	11	0.02
3	University of Virginia	11	0.05
4	University of Nevada	11	0.09
5	University of California, Los Angeles	9	0.03
6	Ohio State University	9	0.02
7	University of California, Irvine	8	0.00
8	Penn State University	8	0.00
9	University of Georgia	8	0.00
10	University of Santa Cruz	8	0.02

### 3.5. Authors analysis

**Figure 7:**
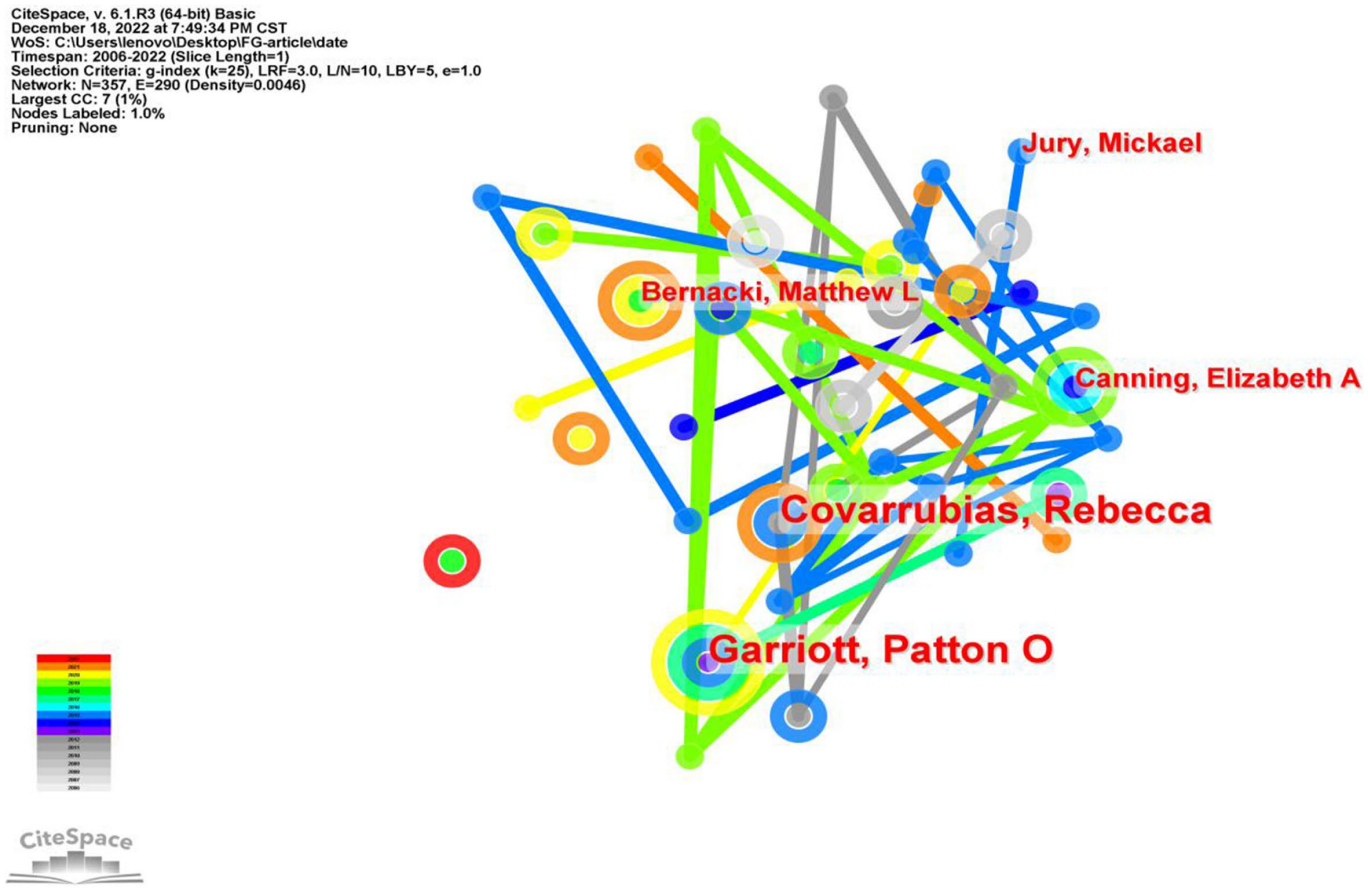
Co-occurrence map of author of FGCS from 2002 to 2022.

We selected articles published between 2002 and 2022 and utilized a time slice of 1 year for our analysis. By choosing authors as the node type in CiteSpace, we generated a co-authorship network map. The merged network consisted of 357 nodes and 290 links, as depicted in Figure 7. Nodes represent authors and lines are used to describe the relationship between authors. The size of the node in the collaboration figure represents the output of the author, the larger the node, the more articles the author has published. The thickness of the line between them reflects the strength of the collaborative relationship between them. Regarding active author, Cavarrubias, Rebeccain from the department of psychology at University of California, Santa Cruz. and Garriott, Patton O from the department of counseling psychology at University of Denver both with 6 publications rank the first. Followed by Jury, Mickael from Universite Clermont Auvergne, Bernacki, Matthew L from the University of North Carolina Chapel Hill and Canning, Elizabeth A from the department of psychology at University of Wisconsin-Madison. The top ten most productive author in this area of study are shown in Table 4. Many of the authors are psychologists by training, and the authors are closely related to each other.

**Table 4:** The top ten author and centrality in the research field of FGCS from 2002 to 2022.

Ranking	Author	Frequency	Centrality
1	Cavarrubias, Rebecca	6	0.09
2	Garriott, Patton O	6	0.01
3	Jury, Mickael	3	0.07
4	Bernacki, Matthew L	3	0.15
5	Canning, Elizabeth A	3	0.02
6	Morales, Danielle X	2	0.04
7	Martin, Julie P	2	0.05
8	Inlelas, Karen Kurotsuchi	2	0.10
9	Darnon, Celine	2	0.07
10	Bantjes, Jason	2	0.09

### 3.6. Cited references analysis

**Figure 8:**
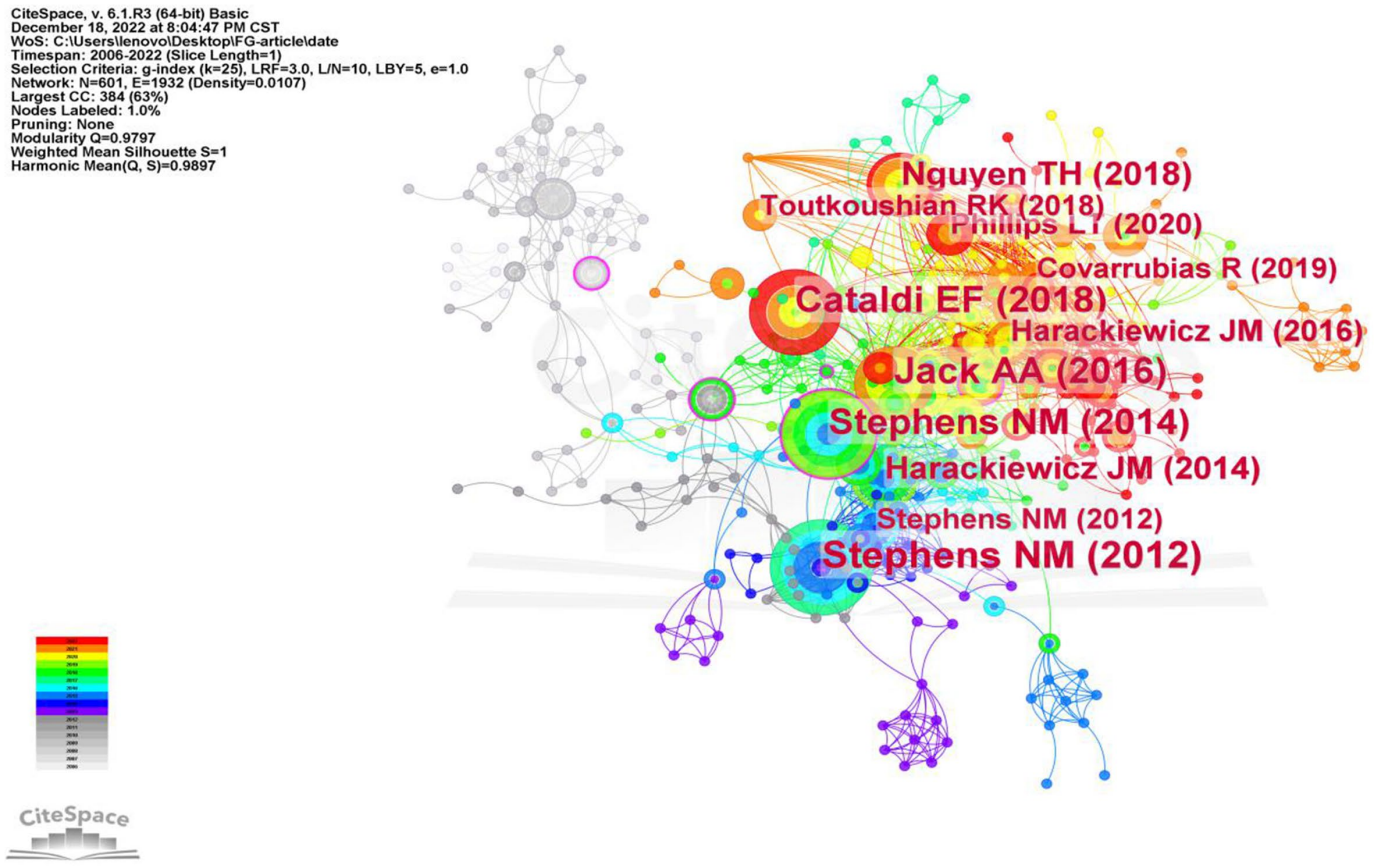
Co-occurrence map of cited references of FGCS from 2002 to 2022.

Co-citation is a research method used to measure the relationships between articles, where two or more articles are cited by one or more papers simultaneously, indicating a co-citation relationship (Zhou et al., 2021). In our analysis, we selected references as the node type in CiteSpace, and set the year of publication span of the articles between 2002 and 2022, with a time slice of 1 year. This generated a co-citation network map, which revealed a merged network comprising 601 nodes and 1932 links, as illustrated in Figure 8. Nodes represent co-citations and lines are used to describe the relationship between co-citations. The top ten most citations references in this film of study are shown in Table 5. Unseen disadvantage: how American universities’ focus on independence undermines the academic performance of first-generation college students (Stephens et al., 2012) written by Stephens NM in 2012 is the most cited literature. Followed by First-Generation Students: College Access, Persistence, and Post bachelor’s Outcomes. Stats in Brief (Cataldi et al., 2018) written by Cataldi in 2018, and then (No) Harm in Asking: Class, Acquired Cultural Capital, and Academic Engagement at an Elite University (Jack, 2016) written by Jack AA in 2016. We can find that in most of the co-citations, it is about the academic performance, academic engagement and achievement of FGCS.

**Table 5:** The top ten cited reference and centrality in the research field of FGCS from 2002 to 2022.

Ranking	Cited reference	Year	Frequency	Centrality
1	Unseen disadvantage: how American universities’ focus on independence undermines the academic performance of first-generation college students	2012	24	0.10
2	First-Generation Students: College Access, Persistence, and Post bachelor’s Outcomes. Stats in Brief. NCES 2018–421.	2018	23	0.01
3	(No) Harm in Asking: Class, Acquired Cultural Capital, and Academic Engagement at an Elite University	2016	22	0.10
4	Closing the social-class achievement gap: a difference-education intervention improves first-generation students’ academic performance and all students’ college transition	2014	21	0.15
5	Is the “First-Generation Student” Term Useful for Understanding Inequality? The Role of Intersectionality in Illuminating the Implications of an Accepted—Yet Unchallenged—Term	2018	15	0.05
6	Closing the Social Class Achievement Gap for First-Generation Students in Undergraduate Biology	2014	14	0.09
7	Closing achievement gaps with a utility-value intervention: Disentangling race and social class	2016	12	0.04
8	Access is not enough: Cultural mismatch persists to limit first-generation students’ opportunities for achievement throughout college	2020	11	0.01
9	A cultural mismatch: Independent cultural norms produce greater increases in cortisol and more negative emotions among first-generation college students	2012	10	0.02
10	“You Never Become Fully Independent”: Family Roles and Independence in First-Generation College Students	2019	10	0.01

### 3.7. Keywords analysis

**Figure 9:**
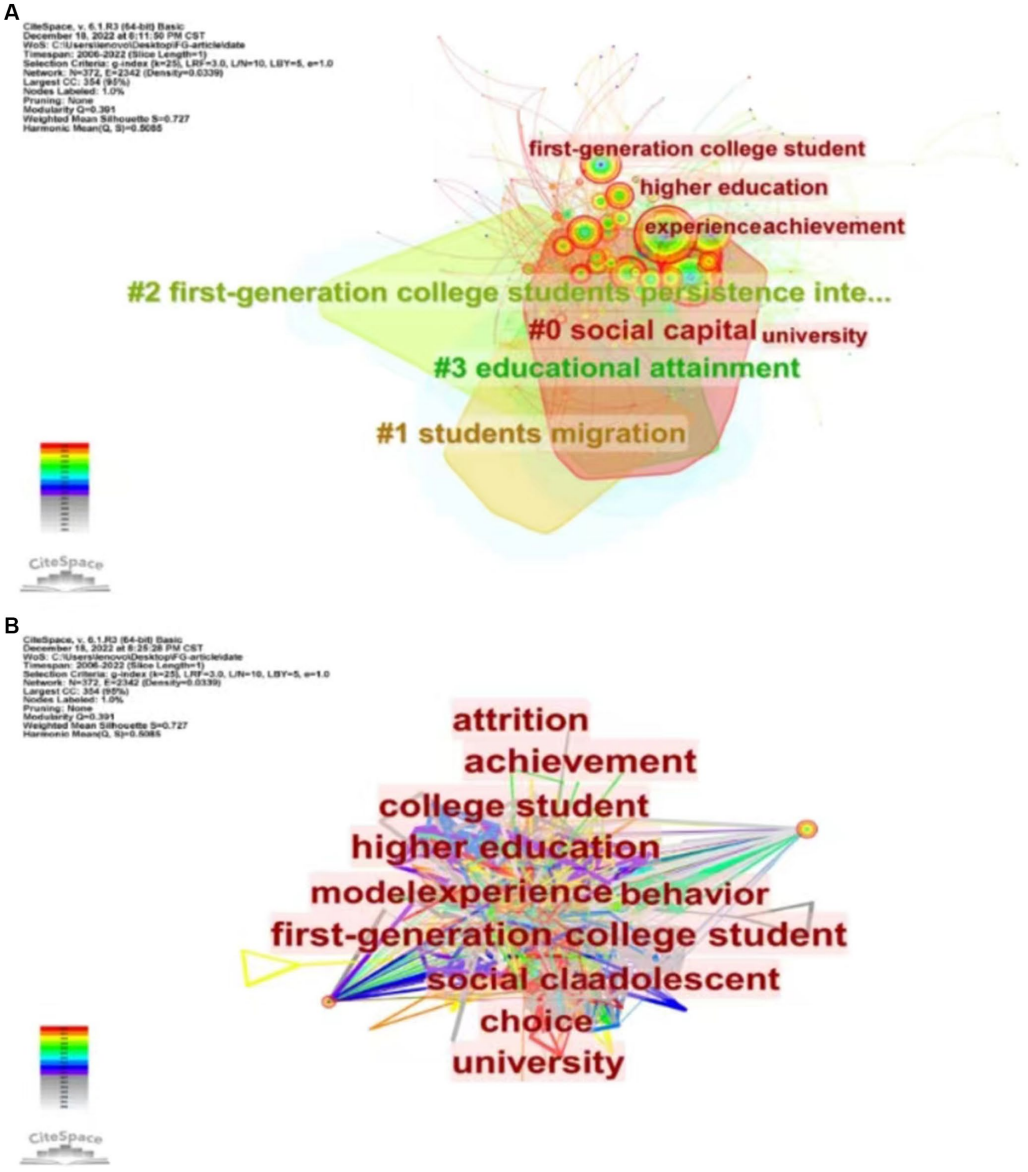
Visual map of keywords of FGCS from 2002 to 2022. **(A)** The clustering map of keywords. **(B)** The co-occurrence map of keywords.

Keywords play a crucial role in understanding the essence of a study. By analyzing keywords, we can summarize the research topics in a specific field, identify hotspots, and explore research directions (Ma et al., 2021). For our analysis, we selected articles published between 2002 and 2022 with a time slice of 1 year, and chose keywords as the node type in CiteSpace. This generated a keyword co-occurrence map, which revealed a merged network consisting of 372 nodes and 2,342 links, as depicted in Figure 9. The keywords that appeared with high frequency in this study are experience (101), higher education (92), first-generation college student (88), college student (64), social class (54), first-generation student (54), student (45), education (44) and achievement (40).

**Figure 10:**
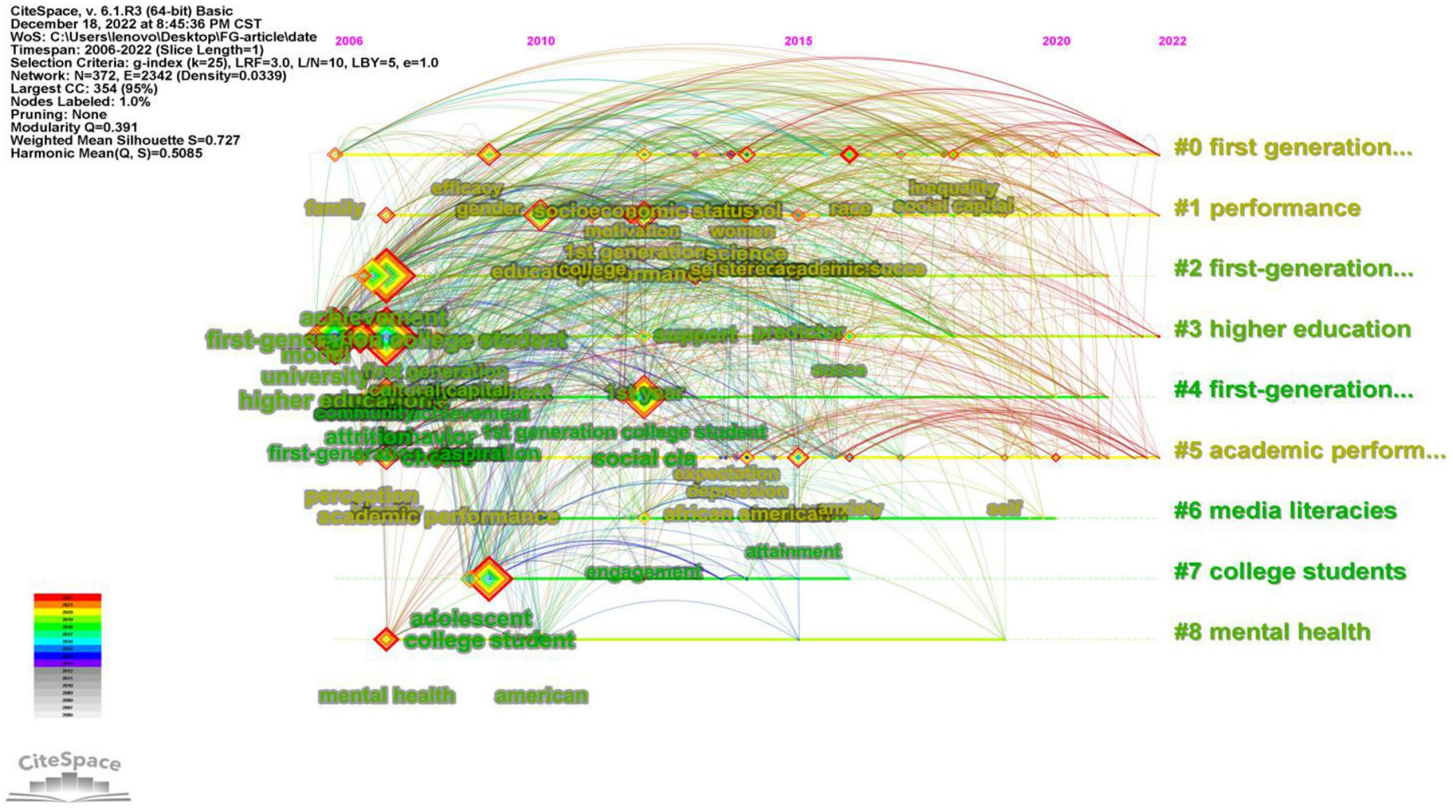
Co-occurrence map of keywords timeline of FGCS from 2002 to 2022.

**Figure 11:**
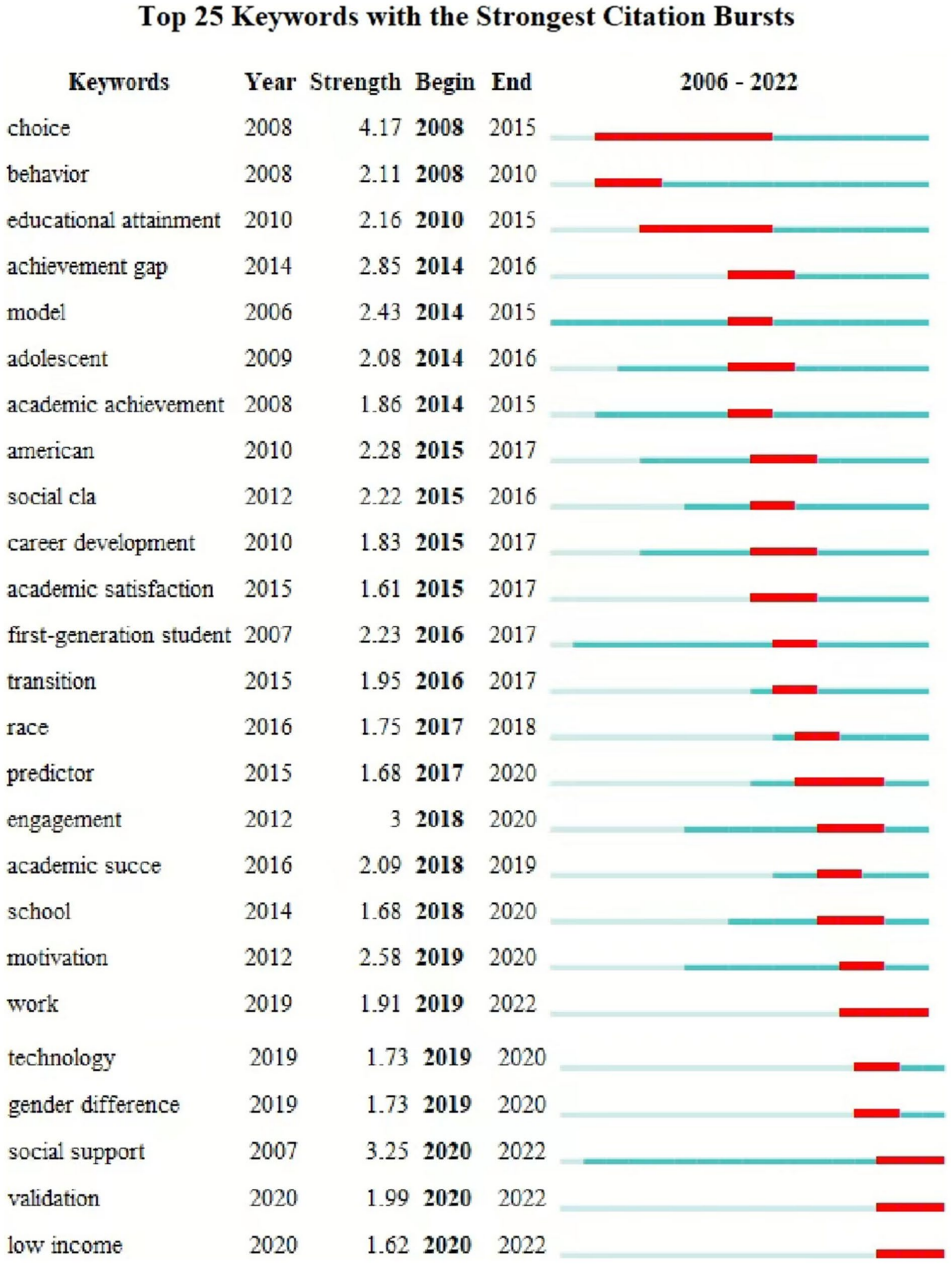
Visualization of top 25 keywords with the strongest citation bursts of FGCS from 2002 to 2022.

The timeline is based on the interactions and mutation relationships between keyword in a given field which helps to explore the evolutionary trajectory and stage characteristics of the research field. Figure 10 is the co-occurrence map of keywords timeline of FGCS from 2002 to 2022 which shows the development directions and hotspots of this field from time dimension Figure 11 shows the top 25 keywords with the strongest citation bursts. The blue line indicates the time intervals and the red line indicates the time of the keyword outbreak. In the field of FGCS researches, choice, behavior and educational attainment are earlier keywords (before 2010), low income, validation and social support are the most recent keywords to appear (after 2020). In addition, choice, educational attainment, predictor, work, social support, academic achievement, achievement gap et al. have a large strength. Race, engagement, school, academic success et al. may be the future trend of FGCS.

## 4. Discussion

### 4.1. Global trends on FGCS

We analyzed and studied the publication, journals, countries, institutions, authors, co-cited references and keywords of FGCS by CiteSpace. The first article on FGCS was *The Gap between Educational Aspirations and Attainment for first-generation College Students and the Role of them Parental Involvement* in McCarron and Inkelas (2006) may be the origin of this theme. This paper examined whether parental involvement has a significant impact on the educational aspirations of FGCS compared to non-first-generation students. In addition, the study examined whether the educational aspirations of FGCS differed from their actual educational attainment. Finally, the study explored differences in educational attainment among first-generation students by gender, race/ethnicity, and socioeconomic status. The growth trend of global FGCS publications from 2002 to 2022 can be divided into two phases. The first phase, 2002–2012 we can call it stagnation or slow emergence phase, the highest number of publications in this decade is 15 in 2012, the total number of publications is 43, which shows its slow. The second phase, 2013–2022 significant growth or rapid development phase, we can see that the number of articles published in each year after 2015 is significantly higher than that of the first phase. Furthermore, 72 publications in 2020, 97 publications in 2021 and 65 publications in 2022 are noteworthy. The total number of publications in this decade is 428, which is about 10 times the number of articles in the first phase.

In general, the highest number of citations per year for a paper usually occurs between 3 and 10 years after publication, and a paper with 100 or more citations is usually considered a “classic” or perhaps even a seminal paper in the field of study (Xiong et al., 2021). The highest number of citations in this field of study is Unseen disadvantage: how American universities’ focus on independence undermines the academic performance of first-generation college students with 531 citations and Closing the social-class achievement gap: a difference-education intervention improves first-generation students’ academic performance and all students’ college transition 309 citations, which are “classic.” Through analysis of classical literature, we find that a growing body of work suggests that teaching FGCS a contextual understanding of difference-that students’ different experiences in college are the result of participating in different contexts before college-can improve their academic performance (Stephens et al., 2014; Townsend et al., 2019). Understanding the root causes of the social class achievement gap in education is an important step in ensuring that education serves its purpose as an engine of social mobility. Dittmann found three main sources of the social class achievement gap—individual skills, structural conditions, and people’s meaning-making processes or ideas. Interventions will be most effective when tailored to the specific needs of students and the context in which they are implemented (Dittmann and Stephens, 2017). Citations analysis show that this emerging field of research is still not to be underestimated, and the papers which published in recent years we collected in this study are expected to gain more attention from researchers in the coming years, and this field is expected to be explored more.

Centrality is a measure of the significance of a node in a co-occurrence graph. It is primarily used to weigh the value of the node’s bridging function in the overall network structure, and usually nodes greater than 0.1 are considered to be relatively important (Song et al., 2022). The result indicate that Research in higher education, Journal of college student development, Journal of higher education and Journal of personality and social psychology are the more influential journals in the field of FGCS. University of Wisconsin, Arizona State University, University of Virginia and University of Nevada are representative institutions. Furthermore, Cavarrubias, Rebecca and Garriott, Patton are highly productive authors, Bernacki, Matthew L (rank 4 in publications) and Inlelas, Karen Kurotsuchi (rank 8 in publications) are authoritative authors, whose centrality greater than 0.1. Most of the authors on the top ten listing are from developed countries, moreover, the United States have the largest number of publications, high-impact journals, academic institutions and authors.

### 4.2. Research focus and hotspots on FGCS

Analysis of the results of high-frequency and strongest burst keywords showed that the research focus and hotspots of FGCS has changed over time. Exploring the behavior, educational attainment and achievement gap of FGCS has been a research hotspot since 2008. Martinez et al. (2009) found that low parental education was associated with a greater risk of college students loss. While college GPA, scholarships, loans, and full-time employment mediate this effect, drug use, psychological distress, and rarely reported academic challenges predict wear and tear unrelated to parental education. These findings may all inform interventions to reduce attrition in college enrollment in the future. Researchers at that time focused their research on performance and decision-making of the FGCS in the field of education, as well as the correlation between their behaviors and choices and educational experience. However, these do not cover the unique difficulties and challenges that FGCS faced, as well as some aspects such as career development and social contribution after completing their university studies. And in recent years, the focus on the relationship between low income, social support, work and academic success with FGCS has gradually increased. The academic issues of FGCS have long been an important topic of research. Phillips et al. (2020) found that an initial cultural mismatch leads to poorer experience and academic achievement for first-generation students, and that these differences persist even after graduation. In addition, first-generation students continued to support more inter-dependence throughout college than continued generation students. In addition, researchers tend to think that the self-pattern of first-generation college students during their entire college years is interdependent, which does not match the independence emphasized by higher education. Therefore, providing opportunity is not enough to reduce social class inequality, universities need to create more diverse cultures and more inclusive environments to ensure that students from different backgrounds receive similar rewards. Mentorship relationships have clearly been shown to promote positive academic and developmental outcomes for young people from diverse backgrounds, which may be a key factor in helping to reduce the FGCS academic opportunity gap (Erickson et al., 2009; Miranda-Chan et al., 2016). Fruiht and Chan (2018) suggest that mentors can act as complementary resources for FGCS, making the academic and retention outcomes of participating FGCS look more like CGCS. So we can add mentor support to the growing list of resources that, when put in place, might create a more level playing field at university for young people from different backgrounds. But FGCS could seldom come into contact with mentors, and mentors cannot fully compensate for lower parental education.

Social class directly affects life satisfaction, academic satisfaction, and GPA, as does first-generation status (Allan et al., 2016). Garriott et al. used hypothetical models to provide an adequate fit to the data with environmental support to predict college self-efficacy, college performance expectations, and academic satisfaction. The results reveal a three-way interaction among academic satisfaction, intrinsic motivation to attend college, and FGCS status to life satisfaction (Garriott et al., 2015). FGCS tend to receive less support for identity development from their mentors compared to CGCS. This lack of support may contribute to lower rates of college enrollment and completion among FGCS. However, community members, relatives, and educators can play a critical role in improving the educational outcomes of FGCS, particularly through mentoring during adolescence. This type of mentoring can provide FGCS with support in goal striving, social skill development, and explicit identity development, which can better moderate the relationship between parental college attendance and educational attainment in adulthood. Instead of relying solely on parental support, the natural mentoring that occurs as FGCS enter and leave college may be a practical and effective way to support their success (Fruiht and Chan, 2018). The identities of FGCS are strongly influenced by their sociocultural context, which in turn shapes their perceptions of personal career paths. These sociocultural factors can serve as potential mediators or even determinants of FGCS’ expectations for their future careers. Understanding and describing the specific factors that influence these expectations is crucial in designing educational experiences that effectively address FGCS’ perceptions. It is important to recognize that well-meaning diversity programs in higher education may not be effective if the unique sociocultural backgrounds of students from underrepresented populations, including FGCS, are not fully understood (Dewsbury et al., 2019). It is crucial for programs that aim to promote diversity and inclusion to evolve and adapt to the changing understanding of diversity. Simply prescribing outcomes related to diversity may not be sufficient. It is necessary to delve deeper into perceptions of diversity and consider the role of families, both current and future, in shaping these perceptions. Academic and social programs at the university and college levels should be inclusive and sensitive to the unique realities and experiences of individuals from diverse backgrounds, including FGCS, in order to effectively support their success.

### 4.3. Study strengths and limitations

Bibliometric analysis can perform visual analysis on bibliographic data, generating knowledge maps and timelines that allow researchers to better understand the relationships and development trends among publications. This study is a prospective study on the bibliometric analysis of FGCS to assess the global trends and prospects in FGCS research over the past two decades. In addition, the literature search terms were specific and comprehensive, and the search was not limited to one academic journal, but used WoSCC as a database to obtain rich data. Furthermore, the bibliometric analysis of this study covered annual publication output, the most cited reference, country, institution and journal distribution, including keyword analysis, popular subject categories, and country and institutional productivity.

Finally, some limitations must be also considered. Firstly, we used only WoSCC data available, excluding other electronic databases such as PubMed or Embase. Therefore, the papers collected from the WoSCC database may be delayed, leading to some bias in the citation counts and H-indexes in the study. Secondly, there may be inherent bias in the citation analysis. Citation rates vary by specialty and depend on the scale of the research field. More popular scientific fields tend to have more classical references. In addition, authors are more likely to cite native language articles, while English language articles are more likely to be cited overall. Thirdly, bibliometric methods do not effectively consider the scientific rigor or validity of publications. Highly cited publications do not necessarily have high scientific quality. In summary, in spite of these limitations, we continue to believe that using CiteSpace for visual statistical analysis can help researchers understand the development trends and research hotspots of publications related to FGCS more comprehensively and deeply.

## 5. Conclusion

Based on the bibliometric analysis of literature related to the FGCS over the past two decades, this study obtained a wealth of effective data to reproduce the research process, explored the global research hotspots and assess the development frontiers of FGCS research while summarizing the past research. It is clearly shown a gradual increase in the number of publications involving FGCS worldwide over the course of 20 years, indicating that FGCS have attained extensive attentions. By analyzing 471 publications, we found that the research in this field has experienced a slow development phase before 2012 and a significant growth phase after 2015, which shows that it is still a young field with broad research prospect. The number of publications has increased from 3 in 2006 to 97 in 2021, which demonstrates a growing richness in this field. The most common research categories are education and social psychology. The United States is the core research force in this area, far outpacing other countries. What is striking is the extensive collaboration between countries, institutions, and authors that have collectively contributed to the development of the FGCS research field. We analyzed the frequency and clustering results of keywords in academic literature showed that the focus and hotspots of FGCS research have changed over time. Some research hotspots are summarized as follows:

1. The main research hotspots before 2010 were on the FGCS’ performance and decision-making in the field of education, exploring the correlation between their behaviors and choices and educational experience. With the deepening of the research, the hotspots were focused on the impacts of social and family background on the academic success and mental health of the FGCS.
2. The academic problems of the FGCS always have been an important topic of research for nearly two decades, and the difficulties and challenges faced by the FGCS in the academic field have attracted much attention.
3. Researchers have begun to place greater emphasis on the social and economic challenges faced by FGCS in recent years, and explore ways to improve their success and self-identification through social support and acceptance.
4. Future research trends may continue to explore the aforementioned aspects, may also focus on the areas of cultural identity, social engagement and career development.

In summary, this study presents a historical, forward-looking perspective and provides a comprehensive and reliable body of research that provides an accurate overview of the global landscape of FGCS research. Although these records do not encompass every single publication on FGCS, they do provide a significant and representative sample that can offer valuable insights into the challenges and opportunities faced by this population. Overall, this research has highlighted the key issues related to FGCS’ academic performance, socioeconomic status, career development, social support systems, and probably trigger more comprehensive and in-depth studies on FGCS.

## Data availability statement

The raw data supporting the conclusions of this article will be made available by the authors, without undue reservation.

## Author contributions

LF, XK, BH, and WS conceived and designed the study. LF and XK conducted the data analysis and drafted the manuscript. WS revised the manuscript. All authors contributed to the article and approved the submitted version.

## Funding

This study was supported by Research on Innovative Path of Sociology Talent Cultivation from the Perspective of New Liberal Arts Education, Jiangxi Province Education Science Planning Project under Grand No. 22YB021.

## Conflict of interest

The authors declare that the research was conducted in the absence of any commercial or financial relationships that could be construed as a potential conflict of interest.

## Publisher’s note

All claims expressed in this article are solely those of the authors and do not necessarily represent those of their affiliated organizations, or those of the publisher, the editors and the reviewers. Any product that may be evaluated in this article, or claim that may be made by its manufacturer, is not guaranteed or endorsed by the publisher.
